# Improved Wound Healing and Skin Regeneration Ability of 3,2′-Dihydroxyflavone-Treated Mesenchymal Stem Cell-Derived Extracellular Vesicles

**DOI:** 10.3390/ijms24086964

**Published:** 2023-04-09

**Authors:** Sehee Kim, Yeokyung Shin, Yujin Choi, Kyung-Min Lim, Yeojin Jeong, Ahmed Abdal Dayem, Yoonjoo Lee, Jongyub An, Kwonwoo Song, Soo Bin Jang, Ssang-Goo Cho

**Affiliations:** 1 Department of Stem Cell & Regenerative Biotechnology and Institute of Advanced Regenerative Science, Konkuk University, 120 Neungdong-ro, Gwangjin-gu, Seoul 05029, Republic of Korea; 2 R&D Team, StemExOne Co., Ltd. 303, Life Science Bldg, 120, Neungdong-ro, Gwangjin-gu, Seoul 05029, Republic of Korea

**Keywords:** MSCs, EVs, flavonoid, anti-inflammation, wound healing, MEK/ERK signal

## Abstract

Flavonoids enhance the self-renewal and differentiation potential of mesenchymal stem cells (MSCs) and have therapeutic activities, including regenerative, anti-oxidative, and anti-inflammatory effects. Recent studies have revealed that MSC-derived extracellular vesicles (MSC-EVs) have therapeutic effects on tissue regeneration and inflammation. To facilitate further research on the therapeutic potential of MSC-EVs derived from flavonoid-treated MSCs, we surveyed the production of EVs and their therapeutic applications in wound regeneration. MSCs treated with flavonoids enhanced EV production twofold compared with naïve MSCs. EVs produced by MSCs treated with flavonoids (Fla-EVs) displayed significant anti-inflammatory and wound-healing effects in vitro. The wound-healing capacity of EVs was mediated by the upregulation of mitogen-activated protein kinase kinase (MEK)/extracellular signal-regulated kinase (ERK) signaling. Interestingly, the protein level of p-ERK under inhibition of MEK signals was maintained in Fla-EV-treated fibroblasts, suggesting that Fla-EVs have a higher therapeutic potential than naïve MSC-EVs (Cont-EVs) in wound healing. Moreover, the in vivo wound closure effect of the Fla-EVs showed significant improvement compared with that of the flavonoid-only treatment group and the Cont-EVs. This study provides a strategy for the efficient production of EVs with superior therapeutic potential using flavonoids.

## 1. Introduction

Wound healing is a dynamic process involving homeostasis, inflammation, proliferation, and remodeling. Each phase is coordinated by the interaction of immune cells, epithelial and mesenchymal cells with growth factors, cytokines, and chemokines [1]. Several studies have revealed that MSCs play pivotal roles in all phases of wound healing [2,3].

Previous studies have reported that the paracrine mechanism of mesenchymal stem cells (MSCs) can exert therapeutic effects via extracellular vesicles (EVs) [4,5]. EVs, which are large and small phospholipid-bilayer vesicles ranging in size from 30 to 150 nm, are secreted by cells [6,7]. These vesicles play a critical role in intercellular communication by carrying cargo such as growth factors, proteins, and genetic molecules derived from parent cells [8,9,10]. The composition and characteristics of EVs depend on their parent cells [11]. For instance, several studies have demonstrated that EVs derived from MSCs possess angiogenic, migratory, and immunomodulatory capacities and can promote tissue regeneration in various diseases [12,13,14]. The remarkable involvement of MSC-EVs in controlling extracellular matrix remodeling, cellular proliferation, inflammatory processes, angiogenesis, and activation of specific signaling pathways is attributed to their wound-healing capacity [15,16]. These benefits of EVs have shown clinical applications in the treatment of various diseases.

Recent studies have revealed that flavonoids stimulate the proliferation and differentiation of MSCs [17,18]. Flavonoids are naturally derived substances in plant parts, such as fruits, leaves, and flowers, that are lipophilic [19]. Flavonoids are polyphenolic structures that depend on their functional groups, with approximately 5000 discovered so far [20]. Flavonoids have been widely investigated because of their antioxidant [21,22,23], anti-inflammatory [24,25], and wound-healing functions [26,27]. Moreover, flavonoids are able to interact with cellular membranes, particularly lipid rafts, which present various membrane proteins, including receptors and channel proteins that regulate cellular signaling pathways and biological functions [28]. The signaling pathways regulated by flavonoids include phosphatidylinositol-3 kinase (PI3K)/protein kinase B (PKB) [29,30], the mitogen-activated protein kinase (MAPK) pathway, and especially the mitogen-activated protein kinase kinase (MEK)/extracellular signal-regulated kinase (ERK) pathway [31].

In our previous study, we screened flavonoids with different positions of the hydroxy group on the B ring and found that 3,2′-dihydroxyflavone (3,2′-DHF) significantly enhanced the proliferation rate and stemness of human induced pluripotent stem cells (iPSCs) [32]. Based on previous reports, we hypothesized that 3,2′-DHF could improve the proliferation of MSCs and activate cellular communication. Therefore, the purpose of this study was to determine whether 3,2′-DHF-treated MSCs could produce therapeutically effective EVs as well as promote the production of EVs. In this study, we investigated the functional effects of EVs produced from 3,2′-DHF-treated MSCs and 3,2′-DHF in an excisional wound-healing model. Moreover, we attempted to determine which signals are related to the wound-healing effect of EVs derived from 3,2′-DHF-treated MSCs.

## 2. Results

### 2.1. Screening Proliferative Effects of Various Flavonoids on MSCs

**Figure 1:**
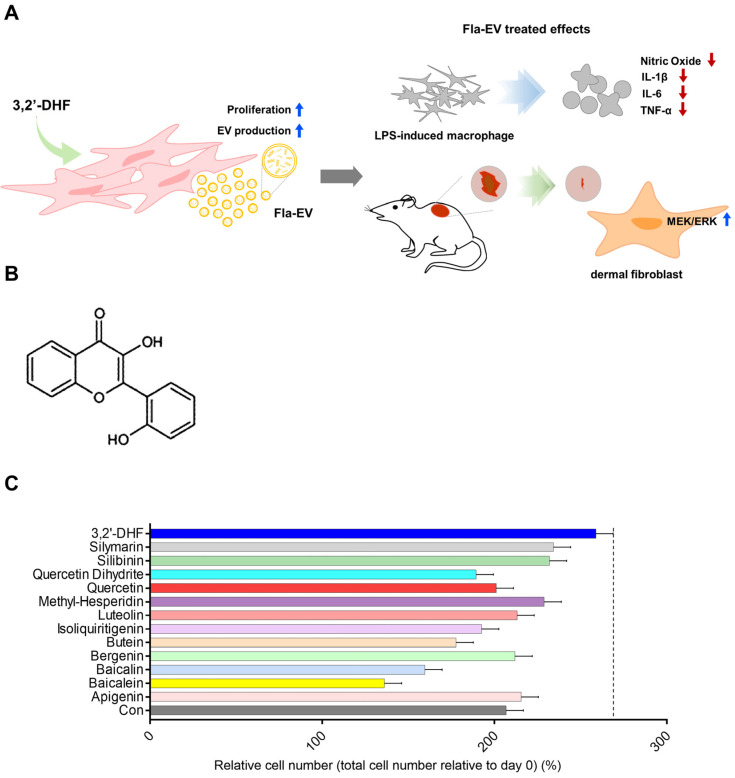
Structure of 3,2′-DHF and screening of several flavonoid treatments in WJ-MSCs. (**A**) Schematic illustration of therapeutic effects of Fla-EV. (**B**) Structure of 3,2′-dihydroxyflavone. (**C**) Comparison of time-dependent cell growth in several flavonoid treatment conditions derived from various sources.

To determine flavonoids that enhance cell proliferation, we examined the growth rate of stem cells with the treatment of various flavonoids such as apigenin, baicalein, baicalin, bergenin, luteolin, methyl hesperidin, quercetin dihydrate, silibinin, silymarin, quercetin, isoliquiritigenin, butein, and 3,2′-dihydroxyflavone (3,2′-DHF). WJ-MSCs treated with 3,2′-DHF showed the highest cell growth among the various flavonoids (Figure 1C).

### 2.2. The Proliferative Effect of 3,2′-DHF on MSCs

**Figure 2:**
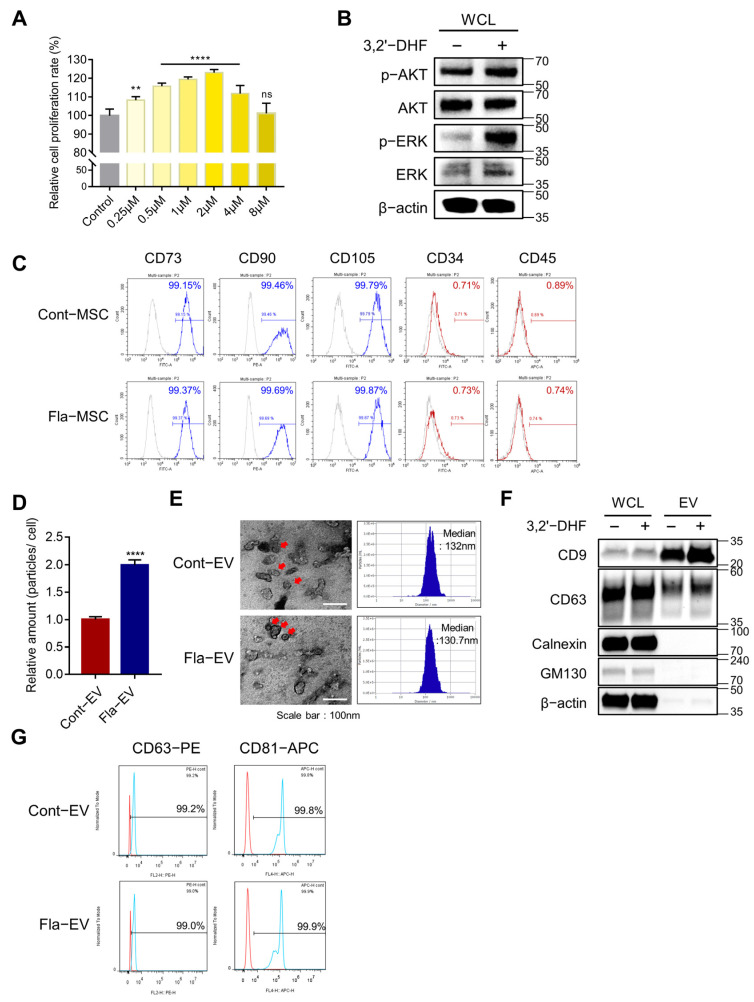
Characterization of 3,2′-DHF-treated WJ-MSCs and EVs. (**A**) The proliferation rate of WJ-MSC treated with various concentrations of 3,2′-DHF. WJ-MSCs have the best proliferation rate with 1–2 μM 3,2′-DHF. Statistical analysis was determined using one-way ANOVA with Tukey’s multiple comparisons test: ** *p* < 0.05, **** *p* < 0.0001. (**B**) Protein expression of MEK/ERK signaling proteins in WJ-MSCs with and without 3,2′-DHF treatment. The bar graph shows the protein expression of AKT and MEK in 3,2′-DHF-treated and untreated WJ-MSCs. (**C**) Flow cytometry analysis to confirm the characteristics of WJ-MSCs with or without 3,2′-DHF. The expression of the positive markers CD73, CD90, and CD105, and the negative markers CD34 and CD45, was assessed. (**D**) The relative EV production amounts of Cont-EV and Fla-EV were measured using nanoparticle tracking analysis (NTA). Statistical analysis was determined using two-way ANOVA: **** *p* < 0.0001. (**E**) Transmission electron microscopy (TEM) images of Cont-EV and Fla-EV. Scale bar: 100 nm. The size of the EVs was determined with NTA. (**F**) Characterization of EV-positive markers CD9 and CD63, and negative markers calnexin and GM130, in Cont-EV and Fla-EV using Western blotting. (**G**) Representative plot of EV-surface markers CD63 and CD81 in Cont-EV and Fla-EV via flow cytometry analysis.

We investigated the effects of several concentration ranges (0.25–8 μM) of 3,2′-DHF on MSCs. To investigate the proliferative effect of 3,2′-DHF, MSCs were incubated with 3,2′-DHF for 48 h. Microscopy images showed that 3,2′-DHF-treated MSCs (Fla-MSC) had no differentiation or abnormality (Supplementary Figure S1). The MTT assay results showed that MSCs treated with 2 μM 3,2′-DHF had a higher proliferation rate than the other groups (Figure 2A). AKT and ERK signals were increased in 3,2′-DHF-treated MSCs at the protein level compared with those of naïve MSCs. The expression levels of phosphorylated AKT (p-AKT) and phosphorylated ERK (p-ERK) were higher than those in the control (Figure 2B). Additionally, we assessed whether MSCs induced by 3,2′-DHF had modified characteristics. The stemness markers CD73, CD90, and CD105 were highly expressed in the control and the 3,2′-DHF-treated MSCs (Figure 2C). Negative markers, such as CD34 and CD45, were expressed in less than 5% of the cell populations in both groups, suggesting that stemness-related markers were maintained under 3,2′-DHF treatment, similar to the naïve condition (Figure 2C). Based on these results, a concentration of 2 μM 3,2′-DHF was used in subsequent experiments.

### 2.3. Characterization and Enhanced Production of EVs from 3,2′-DHF-Treated MSCs

MSCs were cultured with 2 μM 3,2′-DHF, and the supernatant was harvested after 48 h of incubation. The EVs were isolated via conventional differential centrifugation [33]. The EVs expressed tetraspanin markers such as CD63 and CD9; however, calnexin (an endoplasmic reticulum marker) and GM130 (a Golgi marker) were hardly detected in the Western blot results (Figure 2F). The EVs produced with and without 3,2′-DHF were named Fla-EVs and Cont-EVs, respectively. The Fla-EVs (1.11 × 10^3^ particles/cell) had twice the particle number of the Cont-EVs (5.55 × 10^2^ particles/cell) (Figure 2D). Flow cytometry results showed that CD63 and CD81 were expressed in more than 99% of both EV populations, suggesting that the isolated EVs had a higher expression of the tetraspanin marker (Figure 2G). NTA analysis showed that Cont-EVs and Fla-EVs have similar sizes, with diameters of 132 nm and 130.7 nm, respectively (Figure 2E). Negative staining TEM images showed that both EVs had the cup shape of a vesicle (Figure 2E).

### 2.4. Anti-Inflammatory Effects of Fla-EVs

**Figure 3:**
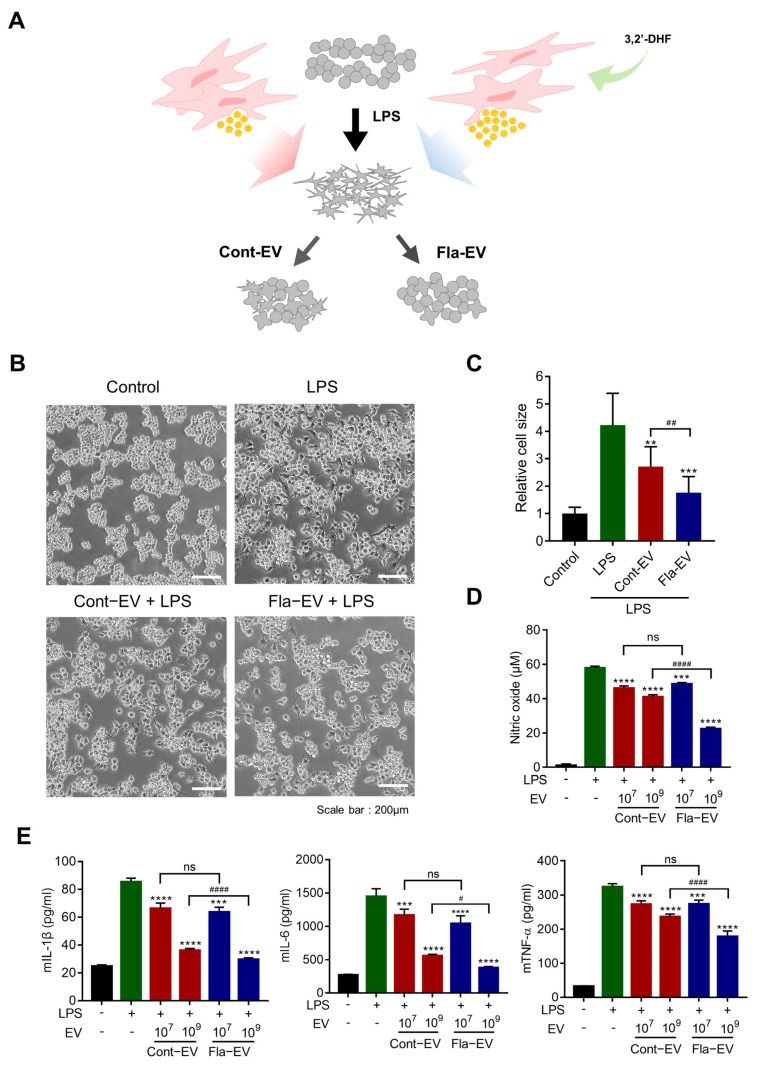
Anti-inflammatory effects of Fla-EVs in RAW 264.7 cells. (**A**) Schematic anti-inflammatory assay of RAW 264.7 cells with LPS and EVs. (**B**) Representative images of RAW 264.7 cells incubated with LPS and EVs for 24 h. Scale bar: 200 μm. (**C**) Relative size of each cell as analyzed with image J. Statistical analysis was determined using one-way ANOVA with Tukey’s multiple comparisons test: ## *p* < 0.01, ** *p* < 0.01, *** *p* < 0.001. (**D**,**E**) RAW 264.7 cells were co-treated with 10 ng/mL of LPS and EVs (1 × 10^7^ and 1 × 10^9^ particles, respectively). (**D**) Nitric oxide levels as determined with the Griess assay. (**E**) Expression level of inflammatory cytokines, including mIL-1β, mIL-6, and mTNF-α, as measured with ELISA. Statistical analysis was determined using one-way ANOVA with Tukey’s multiple comparisons test: # *p* < 0.05, #### *p* < 0.0001, *** *p* < 0.001, **** *p* < 0.0001. # compared with the LPS group and * indicate comparisons between Cont-EV and Fla-EV.

To investigate whether Fla-EVs displayed anti-inflammatory effects, LPS-induced RAW 264.7 cells were used [34]. On exposure to LPS, macrophages acquire a pro-inflammatory phenotype and produce interleukin-1 (IL-1), interleukin-6 (IL-6), TNF-α, and inducible nitric oxide synthase (iNOS) [35]. RAW 264.7 cells stimulated by LPS had irregular shapes compared with the round shape under normal culture conditions. Therefore, morphological changes could indirectly explain the pro-inflammatory or anti-inflammatory phenotype of the RAW 264.7 cells. As shown in Figure 3B, LPS-treated cells displayed changes in shape (irregular form). However, the Cont-EV and Fla-EV treatment groups showed only a smaller proportion of cells whose shape was changed compared with those of the LPS group (Figure 3B). Moreover, the cells had a rounder and smaller shape in the Fla-EV treatment groups compared with the Cont-EV treatment groups (Figure 3B,C).

Nitric oxide (NO) produced by iNOS was measured in the culture supernatant using a colorimetric assay. The levels of proinflammatory cytokines were measured using ELISA. The level of NO significantly decreased in the Cont-EV or Fla-EV treatment groups compared with that in the untreated EV group (Figure 3D). The Fla-EV-treated group showed reduced expression of pro-inflammatory cytokines, including IL-1β, IL-6, and TNF-α (Figure 3E). Remarkably, 1 × 10^9^ particles of Fla-EV-treated cells significantly attenuated NO levels compared with the same particle number in the Cont-EV group (approximately two times) (Figure 3D). The most dramatic decrease in IL-1β, IL-6, and TNF-α levels was observed in 1 × 10^9^ particles of the Fla-EV group (Figure 3E). These results demonstrate that Fla-EVs have a superior anti-inflammatory effect in a dose-dependent manner and are more effective than Cont-EVs.

### 2.5. In Vitro Wound Closure Effect of Fla-EVs on Human Dermal Fibroblasts and Keratinocytes

**Figure 4:**
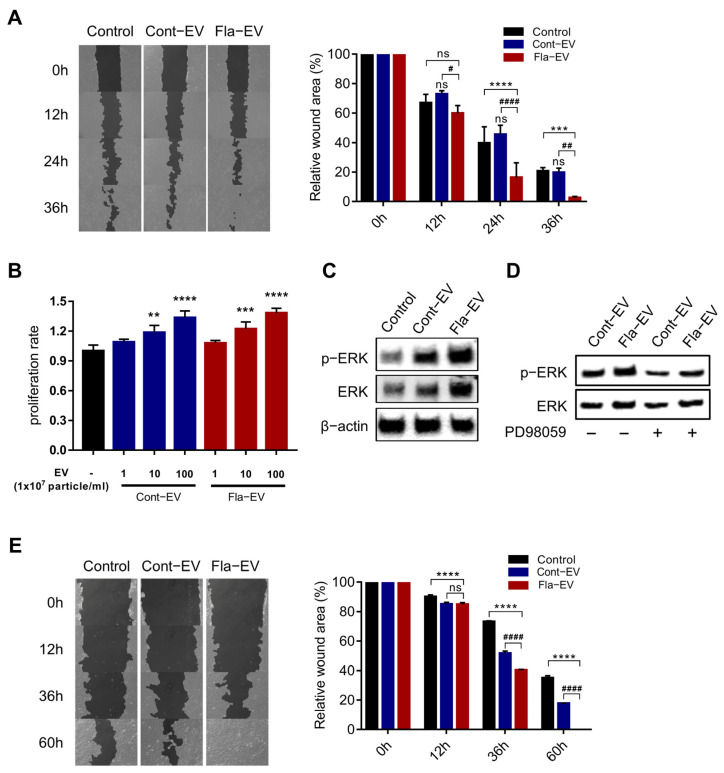
In vitro wound-healing effects of Fla-EVs. (**A**) Representative image of NHDFs incubated with EVs after scratching. The bar graph shows the relative wound area in each group. * indicates comparison with control and # indicates comparison with Cont-EV. Statistical analysis was determined using two-way ANOVA with Tukey’s multiple comparisons test: # *p* < 0.05, ## *p* < 0.01, #### *p* < 0.0001, *** *p* < 0.001, **** *p* < 0.0001. * indicates comparison with control and # indicates comparison with Cont-EV. (**B**) Proliferation rate of NHDFs treated with 1 × 10^7^, 1 × 10^8^, and 1 × 10^9^ EV particles. Statistical analysis was determined using one-way ANOVA with Tukey’s multiple comparisons test: ** *p* < 0.01, *** *p* < 0.001, **** *p* < 0.0001. * indicates comparison with control. (**C**) Western blot image showing p-ERK and ERK expression levels in EV-treated NHDFs. (**D**) Representative Western blot image of EV-treated NHDFs with or without PD98059. (**E**) Representative image of HaCaTs incubated with EVs after scratching. The bar graph shows the relative wound area in each group. Statistical analysis was determined using two-way ANOVA with Tukey’s multiple comparisons test: **####** *p* < 0.0001, **** *p* < 0.0001. * indicates comparison with control and # indicates comparison with Cont-EV.

In this study, we investigated the effect of EVs on human dermal fibroblasts in terms of migration and proliferation. We used a scratch assay to assess the migration effects of EVs on human dermal fibroblasts. The gap created by scratching was closed by cell migration, and pictures were taken every 12 h at the same location to monitor cell migration. The results showed that the migration rate of the Fla-EV-treated group was visibly higher than that of the other groups (Figure 4A). Furthermore, we observed that both Cont-EV and Fla-EV increased the proliferation of fibroblasts (Figure 4B).

Previous studies have reported that stem cell secretomes accelerate the wound-healing process via ERK signaling [36]. So, we pretreated fibroblasts with PD98059 (a MEK/ERK inhibitor) to examine whether Fla-EVs could induce wound closure in fibroblasts via MEK/ERK signaling. Interestingly, upon treatment with PD98059, the inhibition of the p-ERK level in Fla-EV-treated fibroblasts was less than in Cont-EV-treated fibroblasts (Figure 4C,D). These results indicate that the enhanced migration of fibroblasts regulated by MSC-EVs may be dependent on MEK/ERK signaling. Importantly, Fla-EVs could induce the migration of fibroblasts despite inhibiting MEK/ERK signaling conditions, maintaining the p-ERK signal by Fla-EVs.

Moreover, we also observed that HaCaT keratinocytes showed increased migration upon treatment with EVs, and that the wound closure was fastest in the Fla-EV-treated group (Figure 4E). Taken together, these results suggest that Fla-EVs exert a wound-healing effect by promoting the migration and proliferation of dermal fibroblasts and migration of keratinocytes.

### 2.6. Regeneration Effects of Fla-EVs in Excisional Skin Wound Model

**Figure 5:**
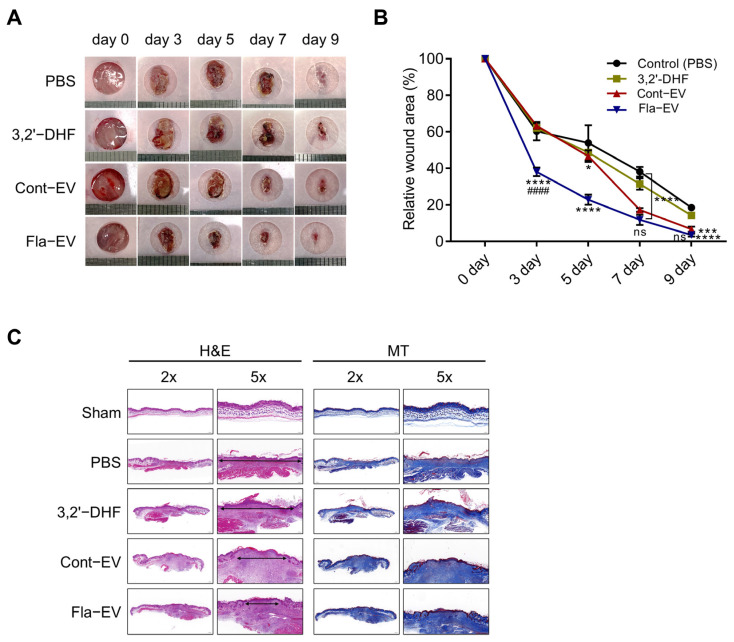
In vivo wound-healing effects of Fla-EVs. (**A**) Representative images of wounds monitored every two days until day 9. (**B**) Graphical data describing relative wound areas calculated to the wound area on day 0 in each group. Statistical analysis was determined using two-way ANOVA with Tukey’s multiple comparisons test: #### *p* < 0.0001, *** *p* < 0.001, **** *p* < 0.0001. * indicates comparison with control and # indicates comparison with Cont-EV. (**C**) Histological analysis of the wound area via hematoxylin and eosin and Masson’s trichrome staining. * *p* < 0.05, *** *p* < 0.001, **** *p* < 0.0001 compared with control; #### *p* < 0.0001 indicates comparison between Cont-EV and Fla-EV.

To assess the in vivo wound-repair capacity of Fla-EVs, we generated excisional wounds in mice by punch biopsy, which is commonly used as a wound model. We acquired images of the wound at five time points within nine days until the new skin completely closed the wound (Figure 5A). Injection of EVs significantly reduced the wound size after five days (n = 3 for each group). Interestingly, the Fla-EV-treated group showed significant wound closure compared with the 3,2′-DHF or Cont-EV groups, suggesting that Fla-EVs have more efficient wound-healing effects (Figure 5A,B). The epithelial gap was evaluated to compare the rate of re-epithelialization in the Fla-EV-treated group. Masson’s trichrome staining was performed to determine the degree of collagen synthesis. The staining results showed that Fla-EV treatment led to significantly improved re-epithelialization in the epithelial layer compared with Cont-EV (Figure 5C).

## 3. Discussion

Many studies have shown that the therapeutic effects of MSCs are primarily induced by paracrine factors through EVs containing various proteins, lipids, and genetic materials, similar to those of parental MSCs, rather than by direct regenerative mechanisms [5,37]. MSC-EV therapy could overcome the concerns about immune responses associated with viable cell transplantation, and the use of MSC-EVs was considered as a cell-free alternative to conventional stem cell therapies, avoiding safety issues such as tumorigenicity [38,39,40]. Despite the cell-free beneficial effects, clinical development is difficult because of low production [41,42,43]. In this study, 3,2′-DHF-treated WJ-MSCs secreted twofold more EVs than naïve WJ-MSCs. Flavonoid-treated MSCs affect various signaling pathways, such as ERK, MEK, and PKB/AKT, to enhance the wound-healing process. Previous studies revealed that flavonoids enhance proliferation and inhibit cellular senescence in human WJ-MSCs [44,45]. In the present study, we found that the EVs produced by 3,2′-DHF-treated MSCs could enhance wound recovery by activating the MEK/ERK pathway. Interestingly, the protein level of p-ERK was maintained in the Fla-EV-treated cells upon inhibition of MEK/ERK, suggesting that Fla-EVs might induce ERK signaling. Studies on the mechanism by which Fla-EVs maintain the expression of p-ERK under conditions of MEK/ERK inhibition require further investigation.

Several recent reports have shown that EV cargo changes based on culture conditions [46,47]. Preconditioning and culture systems can affect the scalable production and therapeutic potential of EVs to stimulate their parent cells [41,48]. We showed that 3,2′-DHF-treated MSCs produced twofold more EVs than MSCs under naïve culture conditions. Moreover, Fla-EVs showed significant anti-inflammatory effects in vitro and displayed higher wound-healing capacity in vivo. This suggests that 3,2′-DHF modulates the physiological effects of MSCs and induces the secretion of more therapeutically potent MSC-EVs compared with naïve MSC-EVs. Further studies are required to discover the EV cargos, including miRNAs, proteins, and lipids, that play key roles in their effects. Moreover, it is necessary to investigate a therapeutic homogenous population of EVs rather than naïve EVs.

However, there are several limitations to this study that should be addressed in further research. First, further studies should investigate the anti-inflammatory effects of Fla-EVs in vivo using animal models of wound healing and inflammation. Our hypothesis was that Fla-EVs may reduce inflammation via the modulation of inflammatory cytokines and infiltration of immune cells, which requires further in vivo validation. While mice primarily heal wounds through contraction, other mechanisms such as inflammation, angiogenesis, re-epithelialization, and collagen deposition also play critical roles in the wound-healing process. It is worth noting, however, that despite the differences in wound-healing mechanisms between mice and humans, many similarities also exist [49]. Therefore, these studies could provide valuable insights into the mechanisms underlying the therapeutic effects of EVs and may lead to the development of new treatments for inflammatory disorders and chronic wounds. Additionally, more application studies to investigate the anti-inflammatory and regenerative effects of Fla-EVs in various in vivo disease models are needed. This would allow us to evaluate the therapeutic potential of Fla-EVs in treating a range of conditions, including chronic wounds, cardiovascular diseases, and autoimmune disorders.

Second, the mechanism of 3,2′-DHF-enhanced EV secretion demonstrated in the present study was related only to the ERK-signaling pathway. Further experiments are needed to investigate the significant changes related to the biogenesis of EV secretion in 3,2′-DHF-treated MSCs. It is important to understand the underlying mechanisms of EV secretion to optimize the therapeutic potential.

Finally, the composition changes in EV cargo must be studied to assess the therapeutic effects of 3,2′-DHF. The cargo of EVs is known to play a critical role in their biological functions [10]. Therefore, understanding the composition changes in EVs after 3,2′-DHF treatment is essential to determine the efficacy and safety of 3,2′-DHF-enhanced EVs as a potential therapeutic agent.

In summary, the current study provides valuable insights into the potential of Fla-EVs as a wound-healing agent. However, further studies are needed to address the limitations of this study and to fully understand the mechanisms underlying the therapeutic effects of EVs. These studies could lead to the development of novel therapeutic strategies for the treatment of inflammatory disorders and chronic wounds.

## 4. Materials and Methods

### 4.1. Cell Culture

Wharton’s jelly MSCs (WJ-MSCs) were isolated as described in our previous report (IRB no. 7001355-202010-BR-407) [50]. WJ-MSCs were cultured in alpha-minimum essential medium (α-MEM, 12561072, Gibco, Waltham, MA, USA) containing 10% fetal bovine serum (FBS, PEAK) and 1% penicillin/streptomycin (PS, 15140-163, Gibco) in 5% CO_2_. RAW 264.7 and normal human dermal fibroblasts (NHDFs) were cultured in Dulbecco’s Modified Eagle Medium high glucose (Capricorn, Ebsdorfergrund, Germany) supplemented with 10% FBS and 1% PS. To determine the effect of MEK/ERK signaling on proliferation, NHDFs were co-cultured with PD98059 (a MEK/ERK inhibitor) at a concentration of 50 μM.

### 4.2. Cell Viability Test

To determine the proper concentration of 3,2′-dihydroxyflavone (3,2′-DHF) in WJ-MSC, 7.5 × 10^3^ cells were seeded in a 48-well cell culture plate (3548, Corning Incorporated Costar, NY, USA). WJ-MSCs were cultured with 1, 2, 4, and 8 μM 3,2′-DHF in α-MEM containing 1% PS without serum. After 24 and 48 h, the medium was replaced with fresh medium containing reagents from the viability assay kit (B1007-500, Cellrix). An xMark™ microplate absorbance spectrophotometer (Table 1; Bio-Rad Laboratories, Hercules, CA, USA) was used to measure cell viability by quantifying absorbance at 450 nm every 30 min.

To screen several flavonoids that enhance cell proliferation, we examined the growth rate of stem cells in the treatment of various flavonoids such as apigenin, baicalein, baicalin, bergenin, butein, luteolin, methyl hesperidin, quercetin dihydrate, silibinin, silymarin, quercetin, isoliquiritigenin, and 3,2′-dihydroxyflavone (3,2′-DHF). All flavonoids were purchased from the INDOFINE Chemical Company (NJ, USA) and dissolved in dimethylsulfoxide (DMSO, SC-358801, Santa Cruz Biotechnology, Dallas, TX, USA).

### 4.3. Isolation and Characterization of EVs

To isolate EVs, WJ-MSCs were seeded at a density of 5000 cells/cm^2^. Upon reaching 80% confluence, the medium was replaced with α-MEM containing 10% EV-depleted FBS and then incubated for 48 h. For the depletion of EVs in FBS, FBS was centrifuged as previously described [51]. To generate Fla-EVs, WJ-MSCs were cultured with 1–2 μM 3,2′-DHF. The cell culture supernatant was harvested and centrifuged at 300× *g* for 10 min. The supernatant was centrifuged at 2000× *g* for 10 min to remove cell debris. Then, the supernatant was centrifuged at 10,000× *g* for 30 min (Table 1; Avanti J-E Centrifuge, Beckman Coulter, Indianapolis, IN, USA), followed by ultracentrifugation at 178,000× *g* for 2 h using a SW32.1 rotor (Table 1; Optima L-90K ultracentrifuge, Beckman Coulter, Indianapolis, IN, USA). The pellet was resuspended in 0.2-μm filtered 1× PBS (10010023, Gibco) and labelled Cont-EV (3,2′-DHF non-treated group) or Fla-EV (3,2′-DHF treated group).

To measure the number and size of EVs, nanoparticle tracking analysis (NTA) was performed using ZetaView (Table 1; TWIN PMX-220, Particle Metrix, Inning am Ammersee, Germany) with a 488 nm laser. The collected data were analyzed using ZetaView 8.04.02 software to obtain the EV size distributions and concentrations. For measurement, all samples were diluted in 0.2-μm filtered PBS (10010023, Gibco).

### 4.4. Western Blot

Whole-cell lysates (WCLs) were isolated by dissolving WJ-MSC in RIPA buffer (LPS solution) with a protease inhibitor cocktail (87786, Invitrogen, Waltham, MA, USA). The amount of protein in the WCLs and EVs was quantified using a bicinchoninic acid analysis kit (23227, Thermo, Waltham, MA, USA) and electrophoresis with 4–12% Bis-Tris Plus gels (NW04125BOX, Invitrogen/NW04122BOX, Invitrogen). The proteins were then transferred to nitrocellulose membranes (IB23001, Invitrogen) and incubated overnight at 4 °C with primary antibodies (1:1000). The membranes were washed with 1× TBST (TLP-118.1, TransLab, Selangor, Malaysia) the next day and the secondary antibody was incubated at room temperature for 2 h. The bands were visualized using ECL reagent (170-5060, Bio-RAD) and measured using Invitrogen™ iBright™ Imagers (Table 1; CL-1000, Invitrogen). The following antibodies were diluted in 1× blocking buffer (TLP-115.1G, Translab): CD9 (ab263023, Abcam, Cambridge, UK), CD63 (ab109201, Abcam), calnexin (2679, Cell Signaling Technology, Danvers, MA, USA), GM130 (12480, Santa Cruz Biotechnology), p-AKT (sc-293125, Santa Cruz Biotechnology), AKT (CSB-PA000855, Cusabio, Houston, TX, USA), p-ERK (CSB-PA000749, Cusabio), ERK (B7074, Tebu-bio, Le Perray, Ile-de-France, France), β-actin (sc-47778, Santa Cruz Biotechnology), HRP-linked anti-rabbit IgG (7074, Santa Cruz Biotechnology), and HRP-linked anti-mouse IgG (7076, Santa Cruz Biotechnology). More details of all antibodies are described in Table 2.

### 4.5. Transmission Electron Microscopy (TEM)

The morphology of EVs was determined using TEM (Table 1; JEM-1010, Nippon Denshi, Tokyo, Japan) at 80 kV as described in our previous report [50]. To prepare TEM samples, EVs were loaded onto a copper mesh grid (FCF300CU, Sigma-Aldrich, St. Louis, MO, USA), and then incubated for 10 min. After absorption using 3M paper, 10 μL of 0.2-μm filtered 3DW (deionized double distilled water) was absorbed after loading onto the grid for 20 s. Next, EVs were stained with 1% phosphotungstic acid (P4006, Sigma) for 45 s and washed three times with 3DW.

### 4.6. Flow Cytometry

To confirm that the characteristics of the WJ-MSCs did not change following treatment with 3,2′-DHF, flow cytometry was used. After WJ-MSCs were cultured with 1–2 μM 3,2′-3,2′-DHF, cells were detached with TrypLE (12563029, Gibco) and centrifuged at 1000 rpm for 3 min. Cell pellets were resuspended in D-phosphate-buffered saline (DPBS) containing 2% FBS and incubated with primary antibodies at 1:200 for 1 h 30 min at 4 °C. After incubation with the primary antibodies, the cell pellets were washed with PBS and centrifuged at 1200 rpm for 5 min, followed by incubation with the secondary antibodies for 1 h 30 min at 4 °C. The following primary and secondary antibodies were used: CD73 (41-0200, Invitrogen), CD90 (AF2067, R&D Systems, Minneapolis, MN, USA), CD105 (MA5-11854, Invitrogen), CD34 (130-108-040, Miltenyi Biotec, Bergisch Gladbach, Germany), CD45 (130-110-771, Miltenyi Biotec), goat anti-mouse IgG (A28175, Invitrogen), and donkey anti-sheep IgG (ab7009, Abcam). The cell pellets were then washed with PBS and centrifuged at 1200 rpm for 5 min. The fluorescence intensity of antibodies in the cells resuspended in DPBS containing 2% FBS was measured using a flow cytometer (Table 1; CytoFLEX, Beckman Coulter, Indianapolis, IN, USA). Additional details of all antibodies are described in Table 3.

### 4.7. RAW 264.7 Cell Anti-Inflammation Assay

RAW 264.7 cells were seeded at 1.5 × 10^5^ cells/well in a 24-well plate (30024, SPL). After 12 h, cells were co-treated with lipopolysaccharide (LPS, L4391-1MG, Sigma) and EVs and DMEM high media containing EV-depleted FBS; 10 ng/mL LPS and 1 × 10^7^ and 1 × 10^9^ particles of EVs were used. After 24 h, the culture supernatant from each group was collected, centrifuged at 10,000× *g* for 3 min, and stored at −80 °C.

### 4.8. Griess Assay

For nitric oxide measurement in LPS-induced RAW 264.7 cell culture supernatant, 0.1% N-(1-naphthyl) ethylenediamine dihydrochloride in DW, and 1% sulfanilamide in 5% phosphoric acid were mixed at a ratio of 1:1. Then, 100 μL of the reagent mixture and 100 μL of the culture supernatant were loaded into a 96-well plate (30096, SPL) and incubated at 25 °C for 10 min. A Bio-RAD x-MarkTM spectrophotometer (Table 1; Bio-Rad Laboratories, Hercules, CA, USA) was used to measure the nitric oxide concentration at an absorbance of 540 nm.

### 4.9. Enzyme-Linked Immunosorbent Assay (ELISA)

ELISA was performed to detect mIL-1β (BGK10749, Peprotech, Cranbury, NJ, USA), mIL-6 (BGK08505, Peprotech), and mTNF-α (BGK06804, Peprotech) in RAW 264.7 cell culture supernatant, following the provided manufacturer’s instructions. The cell culture supernatant was diluted with PBS (10, 50, 100, and 200 times) to confirm cytokine expression levels. According to the test results, the cell supernatant was diluted and loaded onto each well coated with the antibody in advance, and then incubated at 37 °C for 1 h 30 min. Next, 1× biotinylated antibody and 1× avidin-biotin-peroxidase complex were added sequentially. Afterward, the color-developing reagent was loaded and incubated for 10–30 min, and the stop solution was added to terminate the reaction. Finally, a Bio-RAD x-MarkTM spectrophotometer (Table 1; Bio-Rad Laboratories, USA) was used to measure the pro-inflammatory cytokine levels.

### 4.10. In Vitro Migration Assay (Scratch Assay)

NHDF cells were seeded in a 6-well cell culture plate (32006, SPL) at 2.5 × 10^5^ cells/well and incubated for 36 h to reach approximately 100% confluence. After reaching 100% confluence, 10 μg/mL of mitomycin C (M4287, Sigma-Aldrich) diluted in DMEM high glucose (D6429, Sigma-Aldrich) was added per well. After incubation for 2 h, the cells were scratched vertically using a 1000-μL pipet tip (AXG.T-1000-B, Axygen) and incubated with 1 × 10^9^ particles/mL of EVs in same medium. Then, cells were observed at the same positions at 0, 12, 24, and 36 h. TScratch software was used to analyze the migrated area as described in our previous report [50].

HaCaT cells were seeded in a 6-well cell culture plate (32006, SPL) at 6 × 10^5^ cells/well and incubated to reach approximately 100% confluence. Migration assay was performed in the same way as for the NHDF cells. Then, cells were observed at the same positions at 0, 12, 36, and 60 h. TScratch software was used to analyze the migrated area.

### 4.11. Skin Excisional Wound-Healing Mouse Model

Six-week-old immunodeficient BALB/c nude female mice were purchased from ORIENT BIO Animal Center (Seongnam-si, Republic of Korea) and adapted for two weeks. Additionally, the use of immunodeficient mice allowed us to determine the effects of the Fla-EVs on wound healing, without the confounding effects of an intact immune system. While innate immunity plays an important role in wound healing, it can also complicate the interpretation of results, as it involves multiple pathways and cell types [52]. All the experiments were approved by the Institutional Animal Care and Use Committee (IACUC no. KU20132-1) of Konkuk University. The mice were kept under a 12 h light/12 h dark cycle in a well-ventilated room with controlled temperature and humidity for proper adaptation. The animals were provided with food and water ad libitum. After anesthesia by intraperitoneal injection of a 4:1 mixture of Alfaxan and Rompun, all experiments were conducted. To form two identical wounds, the skin on the back of the mouse was excised using an 8-mm biopsy punch (BP-80F, Kai). The EVs (1 × 10^9^ particles) and PBS were injected subcutaneously at two points around each wound as described in our previous report [50]. The size of the wound area was monitored on days 0, 3, 5, 7, and 9 using a 30-cm ruler and silicone with an 8-mm hole and analyzed using ImageJ software.

### 4.12. Histological Analysis

After monitoring the wound area, the mice were sacrificed, and the wound areas were collected and fixed with 4% paraformaldehyde (PFA, Biosesang, Seongnam-si, Republic of Korea). To remove residual PFA, the tissues were washed with PBS. After dehydration using several concentrations of alcohol, paraffin embedding was performed. Then, 4 μm-thick tissue slices were cut perpendicular to the wound surface, and the cut tissues were placed on a precoated slide with 0.1% *w*/*v* poly L-lysine (Sigma, St. Louis, MO, USA). To confirm regeneration of the wound area, hematoxylin and eosin staining was performed. Masson’s trichrome staining was performed to assess the degree of collagen synthesis. Tissue slides were scanned using a digital slide scanner (3D Histech, Budapest, Hungary) to analyze the tissue images as described in our previous report [50].

### 4.13. Statistical Analysis

GraphPad Prism (version 7) software was used for all statistical analyses. All the experiments were independently repeated at least three times. One-way or two-way ANOVA was performed to verify statistical significance. *p* < 0.05 was considered statistically significant; *, **, ***, and **** indicate *p* < 0.05, *p* < 0.01, *p* < 0.001, and *p* < 0.0001, respectively; ns means not significant.

## 5. Conclusions

The 3,2′-DHF-treated MSCs improved EV production, and Fla-EVs showed better anti-inflammatory and migratory effects than Cont-EVs. Fla-EVs can enhance wound-healing capacity by upregulating MEK/ERK signaling. The protein level of p-ERK can be maintained by inhibiting MEK signaling, suggesting that Fla-EVs could play a key role in the wound-healing process. Furthermore, Fla-EVs are expected to provide a better wound recovery effect than 3,2’-DHF treatment alone. This suggests that 3,2′-DHF could modulate the physiological effects of MSCs and enhance the production of better therapeutic effects of MSC-EVs compared with naïve EVs.

## Figures and Tables

**Table 1:** List of instrumentation.

Instrumentation	Product Number	Company
xMark™ microplate absorbance spectrophotometer	681150	Bio-Rad Laboratories
High-speed centrifuge	Avanti J-E	Beckman Coulter
Ultracentrifuge	Optima L-90K	Beckman Coulter
ZetaView	TWIN PMX-220	Particle Metrix
iBright™ Imagers	CL-1000	Invitrogen
Transmission electron microscope	JEM-1010	Nippon Denshi
Flow cytometer	CytoFLEX	Beckman Coulter

**Table 2:** List of antibodies for Western blot.

Antibody	Source	Company (Cat No.)
Recombinant anti-CD9 antibody	rabbit	Abcam (ab263023)
Recombinant anti-CD81 antibody	rabbit	Abcam (ab109201)
Calnexin rabbit mAb	rabbit	Cell signaling technology (2679)
GM130 rabbit mAb	rabbit	Cell signaling technology (12480)
p-Akt1 antibody	mouse	Santa Cruz Biotechnology (sc-293125)
Akt1 polyclonal antibody	rabbit	Cusabio (CSB-PA000855)
Phospho-MAPK3/MAPK1 (T202/Y204) antibody	rabbit	Cusabio (CSB-PA000749)
p44/42 MAP kinase antibody	rabbit	Tebu-bio (B7074)
β-actin antibody	mouse	Santa Cruz Biotechnology (sc-47778)
Anti-rabbit IgG, HRP-linked antibody	goat	Santa Cruz Biotechnology (7074)
Anti-mouse IgG, HRP-linked antibody	horse	Santa Cruz Biotechnology (7076)

**Table 3:** List of antibodies for flow cytometry.

Antibody	Source	Company (Cat No.)
CD73 monoclonal antibody	mouse	Invitrogen (41-0200)
Human/porcine/canine CD90/Thy1 antibody	sheep	R&D Systems (AF2067)
CD105 monoclonal antibody	mouse	Invitrogen (MA5-11854)
CD34 antibody, anti-human	mouse	Miltenyi Biotec (130-108-040)
CD45 antibody, anti-human, REAfinity™	human	Miltenyi Biotec (130-110-771)
Goat anti-mouse IgG (H + L), Superclonal™ recombinant secondary antibody, Alexa Fluor™ 488	goat	Invitrogen (A28175)
Donkey F(ab’)2 anti-sheep IgG H&L (PE) preadsorbed	donkey	Abcam (ab7009)

## Data Availability

Not applicable.

## Supplementary Materials

The following supporting information can be downloaded at: https://www.mdpi.com/article/10.3390/ijms24086964/s1. Refs. [53,54,55,56,57,58] are citied in Supplementary Materials.
