# Supplementary material for: Improved Wound Healing and Skin Regeneration Ability of 3,2′-Dihydroxyflavone-Treated Mesenchymal Stem Cell-Derived Extracellular Vesicles
Source: Int J Mol Sci. 2023 Apr 9;24(8):6964. doi: 10.3390/ijms24086964 (PMC10138514; doi:10.3390/ijms24086964)
Supplement: Supplementary file 1 [file ijms-24-06964-s001.zip › Supplementary Figure_minor revision.pdf]

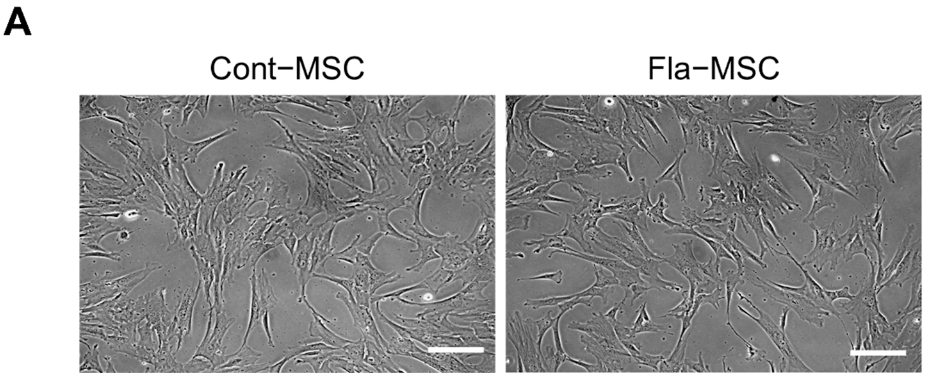

Supplementary Figure S1. Morphology of WJ-MSC upon treatment 3,2′-DHF. The representative cell image of non-treated WJ-MSC and 3, 2′ DHF-treated WJ-MSC. The two groups have similar morphology. Scale bar: 20 μm

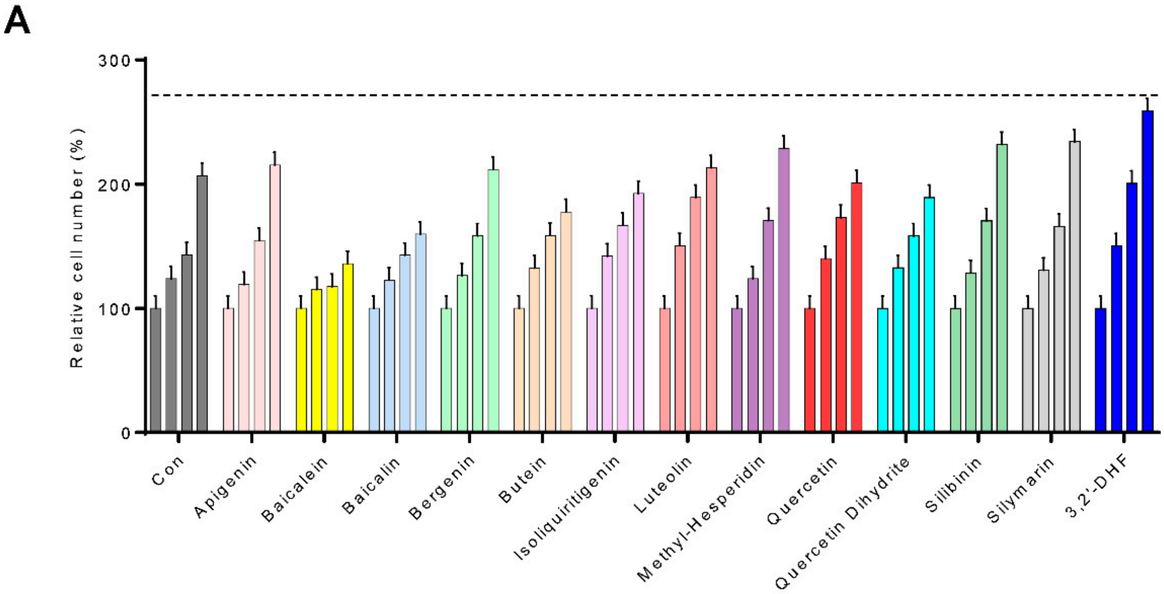

Supplementary Figure S2. Comparison of time-dependent cell growth in several flavonoid conditions. The growth rate of WJ-MSCs treated with several source-derived flavonoids on 24hr, 48hr, and 72hr at the same concentration.
